# A direct analysis method using sheath flow probe electrospray ionisation‐mass spectrometry (sfPESI‐MS) to detect drug residues from fingerprint forensic gel lifts

**DOI:** 10.1002/dta.3688

**Published:** 2024-04-08

**Authors:** Ayoung Kim, Paul F. Kelly, Matthew A. Turner, James C. Reynolds

**Affiliations:** ^1^ Centre for Analytical Science, Department of Chemistry Loughborough University Loughborough UK

**Keywords:** ambient ionisation, direct analysis, fingerprints, forensic analysis, probe electrospray

## Abstract

Latent fingerprints at crime scenes are frequently recovered using forensic gel‐lifters, which can help to preserve the crime scene and to enhance visualisation of traces such as blood or paint. In addition to providing fingerprint ridge detail, additional chemical information can also be recovered from gel lifts that may prove pertinent to an investigation. However, while DNA and metal ions have been shown to be able to be detected in gel‐lifted fingerprints, the determination of other types of chemical information such as the presence of drugs in gel‐lifted prints has not been previously shown. This study demonstrates the application of an ambient ionisation method, sheath flow probe electrospray ionisation‐mass spectrometry (sfPESI‐MS), to the direct analysis of gel‐lifted fingerprints. A model drug compound (zolpidem) is successfully detected from gel‐lifted prints from three different surface types: glass, metal, and paper. The surface activity‐based separation associated with probe electrospray approaches is shown to resolve zolpidem ions from background phthalate species, significantly enhancing the response obtained from the gel‐lifter. A depletion series experiment shows that the drug residue can be detected with up to 100% efficiency after eight consecutive contacts; however, detection efficiency drops to 20% after 30 contacts. The developed approach has potential application to analysis of historical gel‐lifters to obtain additional chemical information.

## Graphical abstract

Sheath‐flow probe electrospray ionisation mass spectrometry is applied to detect drug residues from forensic gel‐lifted latent fingerprints. The sfPESI surface activity‐based separation mechanism helps resolve drug ions from background species improving detection sensitivity. The developed method will have utility in obtaining additional information from historical fingerprint gel lifts.

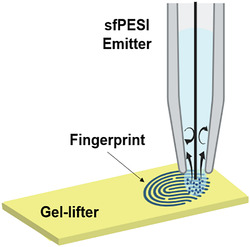

## 1 INTRODUCTION

Fingerprints are one of the most well‐known and utilised forensic sample types; they are particularly useful to investigators for their unique ridge and furrow patterns, which can be used to positively link the fingerprint to an individual.^1^ Individuals' fingerprints develop in utero, and their conformation does not change throughout life, with the exception of growth‐related expansion. Therefore, the fingerprints are distinctive, even for identical twins.^1^ Historically, certain ancient cultures, such as Babylonia and China, employed fingerprints as signatures.^1^ Dactyloscopy, the forensic investigation and comparison of fingerprints as a way of identifying individuals, was first introduced as a reliable method of identification in the 19th century through the pioneering work of Juan Vucetich in Argentina; since then, fingerprints have been a cornerstone of forensic investigations.^1^, ^2^ Fingerprint analysis is essential in modern criminal investigations, and the investigation of barely visible or invisible fingerprints (latent fingerprints), such as those found on glass or paper at crime scenes, have frequently provided crucial criminal evidence. The identification of latent fingerprints at crime scenes typically follows a procedure where latent prints are identified using visual means, which can include alternative light sources (ALS). Latent prints at this stage may often require development to enable a useful fingerprint to be obtained; this can be accomplished using development reagents. There are a wide range of development reagents available, which can involve using chemicals (such as DFO or ninhydrin), powders, or other means^1^, ^3^; once images of finger marks have been obtained, they can then be submitted to databases such as the Integrated Automated Fingerprint Identification System (IAFIS or AFIS), which searches for potential matches.^1^

The technique of lifting latent prints or other relevant marks such as footmarks or paint traces from surfaces at crime scenes using adhesive tape and/or gelatine lifters is well known and offers a number of advantages to crime scene investigators.^4^, ^5^, ^6^ Gel‐lifters are commonly used with prints developed with powders or other development reagents to create an image that will have greater contrast, giving more chance of a successful database identification. The use of gel‐lifters can act to preserve the sample until a digital scan can be taken, and gel‐lifters have the ability to sample relatively large areas providing good coverage of surfaces especially when multiple lifters are used.^4^, ^5^, ^6^ While gel‐lifters are mostly used with already developed prints, they have another advantage in that undeveloped prints can be lifted and then developed on a gel‐lifter, reducing the risk of potential damage to the crime scene and other on‐site evidence or property through the use of latent fingerprint visualisation reagents.^3^, ^4^ For example, Harush‐Brosh et al.^4^ demonstrated that gel‐lifters could be employed as a method for transferring latent prints to the forensic laboratory, which can then be developed in a controlled setting.

Another relatively unexplored capability of gel‐lifters is that they can potentially lift trace amounts of other materials (e.g., drug residues, explosives, and trace ions), which may be invisible to conventional fingerprint visualisation methods but may yet provide information pertinent to the investigation. This material will be extracted onto gel‐lifters with the fingerprints, which after development are stored. Stored gel‐lifters may, therefore, be a source of potentially crucial information. The presence of drugs or drug metabolites in fingerprints could be used to determine whether an individual has been handling drugs or involved in clandestine drug manufacture for example. Several studies^7^, ^8^, ^9^, ^10^ have investigated drug and/or drug metabolite detection from fingerprints; however, each of these studies has looked directly at latent fingerprints and has not attempted to detect drugs from gel‐lifted prints.

Recovery of chemical information from gel‐lifters has been demonstrated in several studies; the most well‐known examples of chemical information being recovered from gel‐lifters relate to the recovery of DNA. Harusch‐Brosh et al. showed that DNA profiles could be recovered from BVDA gel‐lifted finger marks with seven out of 15 finger marks tested reliably identifying the donor, which included determination of the sex of the donor.^4^ Subhani et al. analysed 180 groomed fingerprints, which were developed with four different types of fingerprint powder enhancement before lifting with either fingerprint tape or gel‐lifters. DNA was successfully recovered from all the fingerprints tested. This publication also extended the method to analyse 72 historical fingerprint lifts, enabling DNA profiles to be obtained from 79.2% of historical lifts, which shows the potential of analysing stored evidence to yield further information.^5^ Further work on extracting DNA from gel‐lifts was conducted by Zieger et al., who developed a method for extracting DNA from gelatine lifters following a direct proteolytic digestion, which improved extraction from the gel‐lifter by approximately 25%. This method showed that the majority of DNA from a fingerprint (>80%) is transferred to the gel‐lifter during the lifting process; however, the direct extraction method was seen to only work with the BVDA transparent gel‐lifters as the black and white BVDA lifters were thought to contain inhibitors, which lowered the yield of recovered DNA.^6^

In addition to DNA evidence, gel‐lifters have also been analysed to identify the presence of metal ions, which could potentially be used to link an individual to metal theft. A 2014 study by Bleay et al. used gel‐lifters to take handprint impressions from an individual who had been handling copper or lead objects. The gel‐lifters were then treated with rubeanic acid, which initiated a colour change that showed the presence of the metal ions transferred from the objects and yielded information about the shape of the objects themselves.^11^ Rubeanic acid development of gel‐lifters was later used by Davis et al. to visualise fingerprints from polymer banknotes, a surface that had proved problematic for existing fingerprint development methods. In this study, polymer banknotes were treated with copper using vacuum metal deposition, and then following deposition, gel‐lifters were used to lift the copper‐doped prints before visualisation on the lifter using rubeanic acid.^12^ Other reagents and mechanisms have also been explored in conjunction with gel‐lifted fingerprints; in a recent paper by King et al., the RECOVER fingerprint development system using disulfur dinitride (S_2_N_2_) fuming was used to visualise finger marks lifted from a range of surfaces including copper, steel, glass, and paper; a key finding of this study was that it was possible to tell when fingerprints had been deposited on top of printed prose or vice versa.^13^

All these studies show that a significant amount of material from a latent print is transferred to the lifter, which can then be imaged, developed, or undergo DNA analysis. However, to date, there have been no studies that have looked at sampling other chemical information such as the presence of drugs or other illicit compounds from gel‐lifters. Ambient ionisation mass spectrometry methods offer several advantages for obtaining additional information from forensic samples^14^, ^15^; they can be sampled directly with little or no additional sample preparation, and many ambient ionisation methods are minimally destructive to the sample enabling further analysis to be conducted.^16^ However, a major drawback of most ambient ionisation methods is that there is no separation of analytes between the sampled surface on the mass spectrometer, so issues with ion suppression can arise, which reduces their applicability: this has been particularly problematic with gel‐lifters, which give off high levels of chemical background. Several approaches have been employed to mitigate ion suppression in ambient ionisation methods. In ambient ionisation methods using a liquid extraction, careful optimisation of the extraction solvent can be used to favourably extract analytes and discriminate against the background matrix. Lin et al. demonstrated that by optimising the extraction solvent and the time over which an extraction takes place, improved mass spectra for lipids could be obtained in liquid extraction ambient ionisation.^17^ Using desorption electrospray (DESI), Miao et al. showed that when analysing methamphetamine at low concentrations in a urine sample, increasingly the amount of organic solvent in the DESI spray reduced suppression due to salts in the urine, indicating that the DESI mechanism relies on droplet pickup and by reducing the solubility of salts in the DESI solvent suppression can be mitigated.^18^ The enhancement of analyte responses in thermally desorbed explosives at trace levels from swabs using secondary electrospray ionisation (SESI) was demonstrated by Burns et al.^19^ This research showed that by adding chemical modifiers into the electrospray solvent, the ion chemistry in the ion source could be controlled to promote the formation of specific adduct ions, which improved sensitivity and specificity, with the addition of ammonium chloride providing the best all round performance. Chemical derivatisation of the analytes into a more readily detected form is another way in which ion suppression effects can be mitigated. Bag et al. used an in situ derivatisation of aldehydes with 4‐amino‐phenol to convert them into iminium ions, which dramatically improved their mass spectral responses when analysed with paperspray ionisation‐mass spectrometry.^20^

Another alternative approach is sheath flow probe electrospray ionisation–mass spectrometry (sfPESI‐MS), which is an ambient ionisation technique that has a number of advantages when performing direct analysis from complex matrices.^21^, ^22^, ^23^, ^24^, ^25^, ^26^ sfPESI enables the sampling of a small (<1 mm^2^) area of a latent print by touching an electrospray emitter filled with an extraction solvent (typically ethanol/water) to the sample. A small volume of sample is extracted into the emitter from the surface by the extraction solvent, because only a small area is sampled, the approach is minimally destructive to the surface and multiple extractions and analyses can be performed from a single sample. Once the sample has been extracted, a high voltage is applied to the emitter to generate an electrospray, which ionises the sample. In addition, probe electrospray‐based methods benefit from a rapid surface activity‐based separation, which can help to mitigate matrix interferences and in‐source ion suppression.^25^ This method has been previously applied to quantify cocaine metabolites in dried blood spots where it showed the ability to recover these compounds from a complicated matrix at physiologically relevant levels.^26^ The research presented here applies sfPESI‐MS to determine whether a drug compound can be detected directly from gel lifts of latent fingerprints.

## 2 METHODS AND MATERIALS

Glass microscope slides (W: 25.4 × L: 76.2 × T: 1.2 mm, Sail Brand, China), filter paper (Fisherbrand grade 601, Fisher Scientific, Loughborough, UK), and thin brass plate (W: 20.0 × L: 100.0 × T: 1.0 mm, Sweetnam and Bradley, Malmesbury, UK) were utilised as substrates for deposition of fingerprints; 10‐mg zolpidem tablets (Stilnox, SANOFI, France) were used as a model drug compound to evaluate recovery from gel‐lifted prints; for doping fingerprints, a single tablet (~125 mg) was ground in a mortar and pestle until it became a fine powder. Two different brands of White Gel‐lifters were investigated (BVDA International, Netherlands and Crime Scene Investigation Equipment Ltd, UK) to lift latent finger marks from surfaces in this study. Gel‐lifter sheets were cut to a size of 20 × 60 mm before being used to lift individual latent fingerprints. The transparent plastic cover of the lifter was left in place prior to being used to prevent external contamination before fingerprint lifting. The solvents used in the sfPESI extraction solvent were HPLC‐grade methanol (Fisher Scientific, UK), HPLC‐grade water, and 99.98% absolute ethanol absolute (VWR Chemicals, Lutterworth, UK). The chemical modifiers in the extraction solvent, 95% formic acid and 99% sodium acetate, were both purchased from Sigma‐Aldrich (Gillingham, UK).

### 2.1 Preparation of fingerprints

**FIGURE 1:**
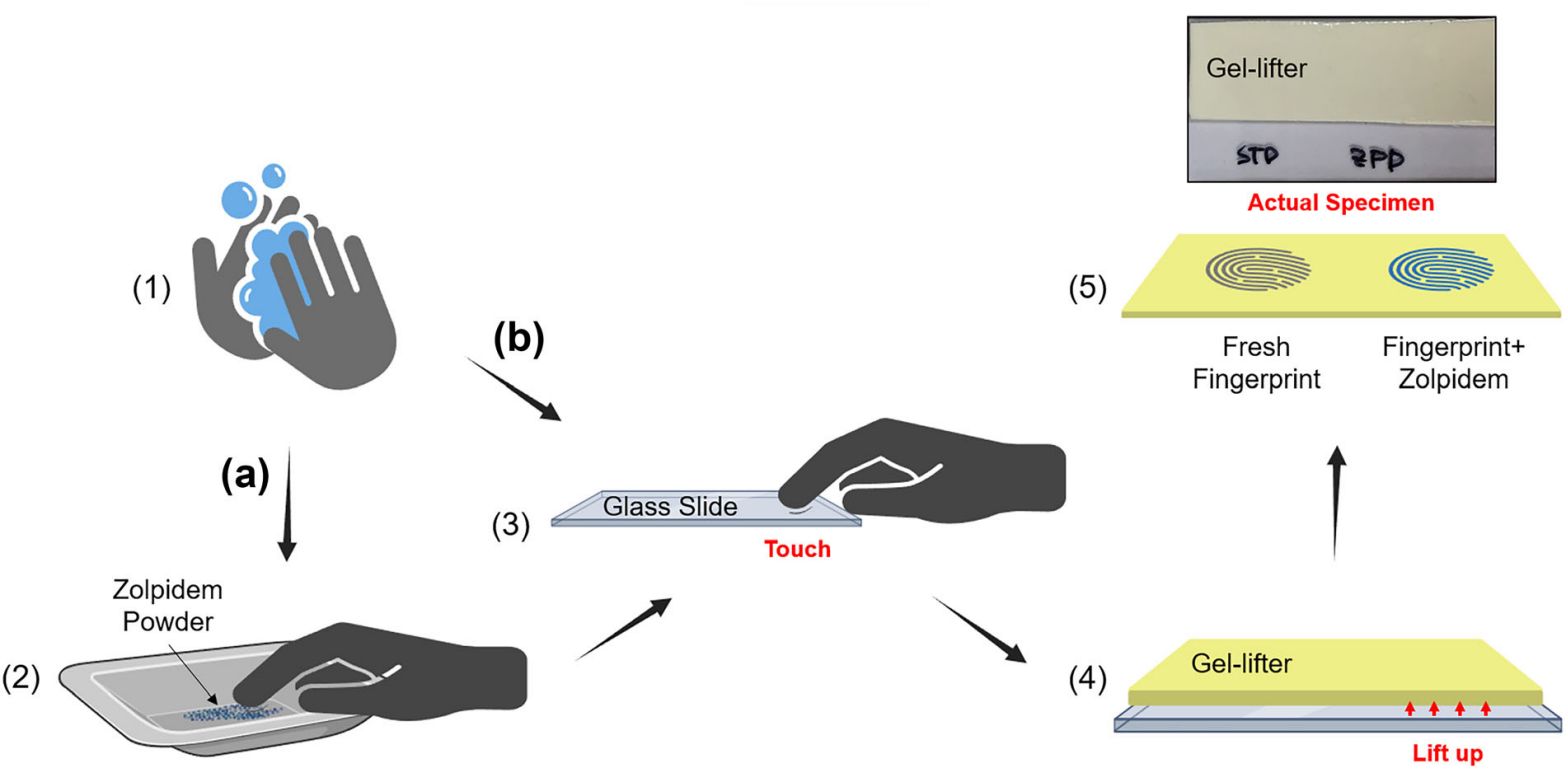
Test sample preparation of (a) ZPD doped fingerprint and (b) fresh fingerprint: (1) wash and dry hands, put on nitrile gloves, wear for 1 h, turn inside‐out and put back on; (2) gently touch ZPD powder with a gloved finger; (3) generate a fingermark onto a glass slide containing (a) ZPD residue or (b) no ZPD residue; (4) take‐up the fingerprint from the slide surface using a piece of gel‐lifter; and (5) store the gel‐lifter piece in a dark place at room temperature (22 ± 1°C) (created with Biorender.com).

Latent fingerprints containing zolpidem residue were deposited on glass, brass, and paper surfaces using the following procedure outlined in Figure 1. To prevent transdermal uptake of zolpidem by the researcher, Microflex 93‐843 nitrile gloves (Fisher Scientific, Loughborough UK) were worn to deposit a fingerprint on the inside of the glove, which was worn for 1 h and then turned inside‐out and replaced leaving the fingerprint on the tip of the glove.^27^ Blank latent prints were taken by depositing a print immediately after putting on the inside‐out gloves: (1) wash hands with soap carefully to reduce undesired interferences from the hands, put on nitrile gloves and wear for 1 h to generate prints on inside of glove, after 1 h, take off gloves, turn inside‐out and put back on; (2) gently touch zolpidem (ZPD) powder with a gloved finger; (3) generate a finger mark containing ZPD residue on the surface; (4) take up the fingerprint from the surface using a piece of gel‐lifter; and (5) store the gel‐lifter containing the fingerprint in a dark place at room temperature (22 ± 1°C) until it is used for sfPESI‐MS analysis. This procedure was intended to avoid finger mark specimens from becoming contaminated with unpredictable environmental contamination and to minimise risk to the researcher while providing similar levels of interference to when fingerprints are deposited directly on a surface. Before touching the surface to generate finger marks, the powder was gently brushed off twice from the contaminated finger until no obvious lumps of ZPD residue were visible on the finger surface.

### 2.2 sfPESI‐MS method

**FIGURE 2:**
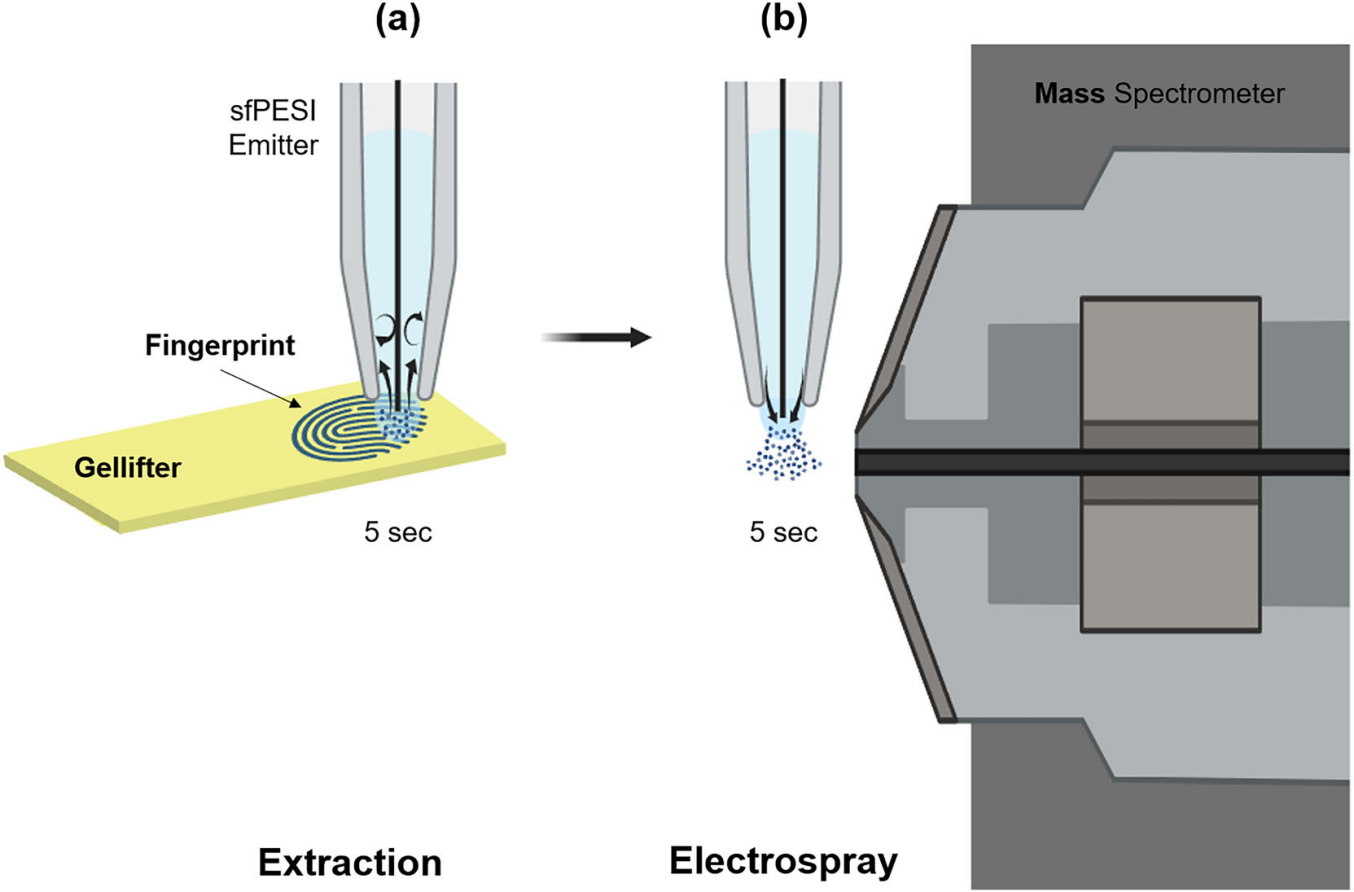
sfPESI‐MS sampling and analysis (not to scale) (a) for 5 s, extract analytes (zolpidem) from the fingerprint by contacting the liquid from the sfPESI probe with the gel‐lifter surface, and then immediately (b) facilitate an electrospray from the sfPESI probe by applying a high voltage of 2.5 kV for 5 s (created with Biorender.com).

All sfPESI experiments were conducted using a Thermo Exactive Orbitrap mass spectrometer (ThermoFisher Scientific, Bremen Germany). The sfPESI emitter was fabricated using a gel‐loader pipette tip (0.5‐ to 20‐μL GELoader tip, Eppendorf AG, Hamburg, Germany) and a fine stainless steel acupuncture needle (J type OD 0.12 mm × L 30 mm, Seirin, Shizuoka, Japan), which is inserted into the gel‐loader tip. The gel‐loading tip was filled with approximately 30‐μL extraction solvent. The tip was connected to a syringe driver (11 Plus, Harvard Apparatus, USA) to enable the emitter to be refilled without having to reposition it. The acupuncture needle was held in place by a silicone septum (11‐mm Non‐Stick BTO septa, Restek, UK) at the top of the gel‐loading tip and protrudes by approximately 0.1 mm from the gel‐loading tip.^22^ The sfPESI emitter was positioned vertically in front of an inlet (2 mm above and 3 mm in front of the inlet) of an Orbitrap mass spectrometer. The sfPESI extraction solvent consisted of a 50% v/v ethanol/water solution, 0.5‐mM sodium acetate, and 0.1% formic acid.^26^ To extract analytes from fingerprints, the sfPESI probe was touched to the surfaces of the gel‐lifted finger mark for 5 s, as shown in Figure 2a. A fixed onset voltage of 2.5 kV was supplied to the sfPESI emitter through an external power supply (292R High Voltage Power Supply, Brandenburg, UK) to allow an electrospray to emit from the probe for approximately 5 s immediately after the conclusion of the surface sampling process (Figure 2b).

A 10 μg/mL zolpidem standard solution was used in direct liquid‐infusion electrospray ionisation (DI‐ESI) for tuning the orbitrap mass spectrometer prior to sfPESI analysis. sfPESI source conditions were further optimised using this solution to provide the optimised conditions shown in Table 1, which were used in all subsequent experiments. Mass spectral data were analysed with a <5 ppm mass error, and the average noise level of each mass spectrum was approximately 7000 c/s by absolute abundance. The scan mass range of the orbitrap mass spectrometer used was *m/z* 50–750 for ZPD analysis in the initial zolpidem recovery experiments and in the analysis of different surface types. A mass range of *m/z* 50–450 was used for the fingerprint depletion series experiments. In all experiments, the scan rate used was 2.4 scans/s.

**TABLE 1:** Optimised sfPESI‐MS conditions for analysis of gel‐lifted fingerprints.

Parameters		Values	Units
Scan	Mass range	50–750	*m/z* ^a^
	Resolution	25,000	‐
	Micro scans	2	‐
	Maximum injection time	10	ms^b^
Ion source^c^	Gas flow rate (Aux, Sheath, Sweep gas)	0	Arbitrary units
	Polarity	Positive	‐
	Spray voltage	2.5	│kV│^d^
	Capillary temperature	300	°C
	Capillary voltage	32.5	V^e^
	Tube lens voltage	90.0	V
	Skimmer voltage	40.0	V

^a^ m/z: mass‐to‐charge ratio.

^b^ ms: milliseconds.

^c^ Ion source: sheath‐flow probe electrospray ionisation (sfPESI) source.

^d^ │kV│: kilovolts in absolute value.

^e^ V: volts.

### 2.3 Preparation of fingerprints for sfPESI different surface types

The properties of the surface upon which a fingerprint is deposited can be critical in crime scene investigation and is known to influence how well analytes can be recovered.^1^, ^3^ Each surface will have its own unique features, and development process utilised to visualise latent fingerprints will differ depending on the surface upon which they are deposited on.^3^ Surfaces are either porous or nonporous. Nonporous surfaces like glass, plastic, or metals will interact with the fingerprint in a very different way to a porous surface like fabrics where the material from the print can permeate into the surface and potentially bind to it. To investigate the effect of surface composition on the sfPESI‐MS recovery of zolpidem, three different surface materials (glass, metal [brass], and paper) were investigated. A single ZPD doped fingerprint was deposited on each surface and then lifted. The individual gel‐lifted prints then had 10 replicate sfPESI extractions and measurements performed on each of them.

### 2.4 Preparation of a fingerprint depletion series

To indicate how long zolpidem persists on the fingerprints and give an indication of whether the technique can detect zolpidem after multiple physical interactions between surfaces and fingers, a depletion series experiment was performed. The ability to detect a drug compound as it depletes through multiple depositions is information that is potentially useful in an investigation to help to explain the activity and behaviour of both criminals and victims using fingerprint evidence. The fingerprints prepared for the depletion series experiment were analysed directly from glass slides. The procedure followed was similar to that described above; however, a further 29 consecutive prints were deposited on individual glass slides (method shown in Figure S1). A fresh sfPESI emitter was used for each print sampled to ensure carryover between prints did not result in a false positive.

## 3 RESULTS AND DISCUSSION

Initial analysis of zolpidem‐doped fingerprints from the gel‐lifters showed very high levels of background interference when averaged mass spectra across the full sfPESI‐MS analysis were examined (Figure S2). This mass spectrum is dominated by several background ions; putative identification of these species is based on accurate mass data. The base peak is an ion at *m/z* 413.2687 (4.8‐ppm error), which corresponds to a sodiated dioctyl phthalate ion with the protonated and potassiated dioctyl phthalate ions detected at lower intensity at *m/z* 391.2867 (4.8‐ppm error) and 429.2427 (4.7‐ppm error), respectively. This species is a plasticiser, which originates either from the gel itself or from transfer from the plastic protective film. A second large peak at *m/z* 115.0375 is also present, and then a range of other species is detected at lower intensity across the mass range between *m/z* 50 and 500, which includes other known plasticisers such as phthalic anhydride, which is detected as an [M + H]^+^ ion at *m/z* 149.0243 (2.7‐ppm error), which can be seen more clearly when zooming in to approximately 10% of the full scale. These data highlight the challenges that occur when analysing directly from gel‐lifters using an ambient ionisation method. The presence of species like phthalates at high levels in the gel‐lifters will dominate the spectrum making visualisation of the target analytes difficult and potentially suppress the formation of analyte ions reducing instrumental sensitivity.

**FIGURE 3:**
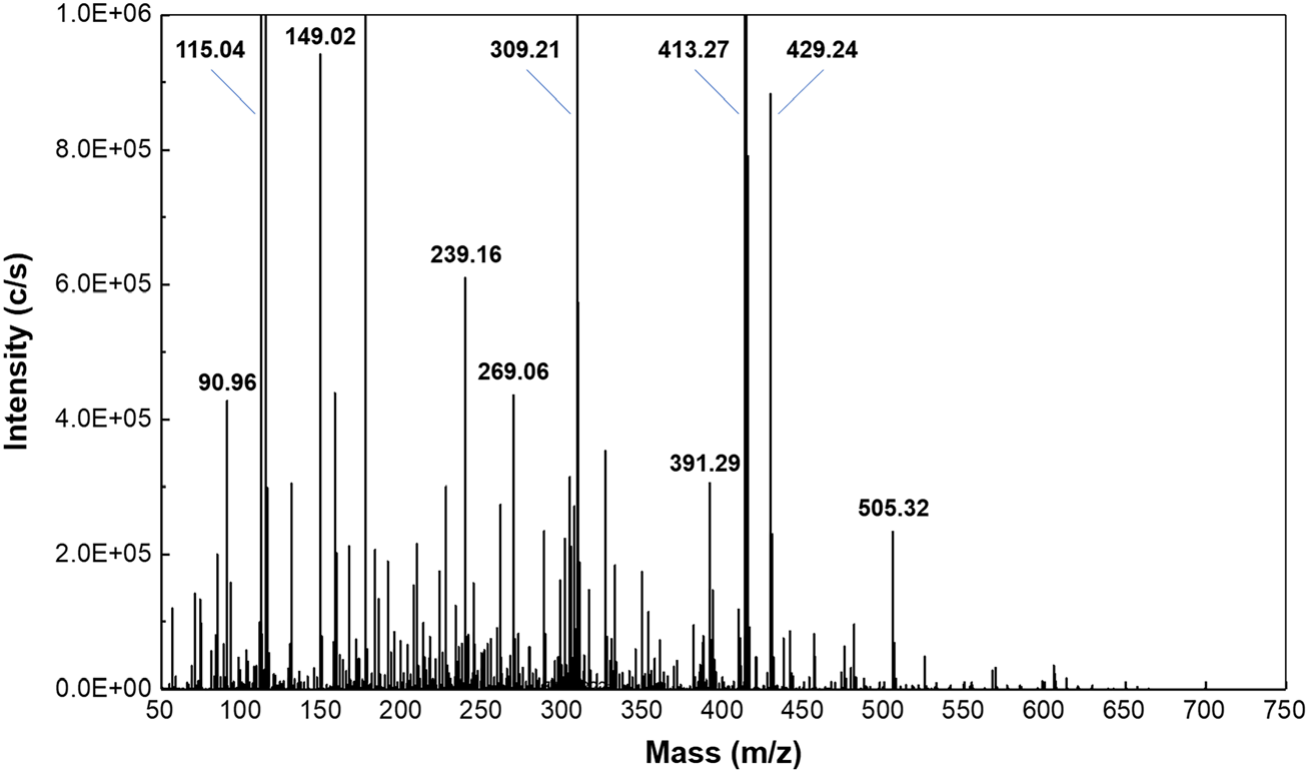
An averaged mass spectrum from the sfPESI‐MS analysis of a gel‐lifted zolpidem‐doped fingerprint (zoomed in approximately 10×).

The sequential ionisation mechanism demonstrated by sfPESI offers a potential rapid solution to mitigate this issue by using a surface activity‐based mechanism to resolve analytes from high‐level background interferences. Figure 3a shows an example of a total ion chromatogram from a single sfPESI‐MS analysis of a zolpidem‐doped fingerprint. The analysis method is rapid with the entire analysis occurring over a timescale of approximately 6 s. Three scans taken at different time points across the analysis are indicated on this scan, and mass spectra obtained at each point are shown. At point S1 immediately after application of the onset voltage, the sodiated zolpidem [ZPD + Na]^+^ ion is detected at *m/z* 330.1572 (3.1‐ppm error) with a less intense peak for the [ZPD + H]^+^ ion detected at *m/z* 308.1755 (2.7‐ppm error), which can be seen in Figure 3a's inset window. The predominance of the [ZPD + Na]^+^ is not surprising as the extraction solvent contains sodium acetate. In addition, there are many eccrine sweat glands on the fingertip ridges, and the sweat they release is high in salt with sodium and chloride ions both being major inorganic components of eccrine sweat with concentrations estimated as 3.3 g/L eccrine sweat (Sodium) and 3.5 g/L eccrine sweat (chloride).^28^ Adding an excess of sodium into the extraction solvent will reduce the effect of variations in the amount of sodium extracted from the surface, which is expected to enhance reproducibility between analyses.^26^ Also evident in Figure 3b is the absence of peaks, which correspond to the dioctyl phthalate or phthalic anhydride interferent ions. The zolpidem ions are then rapidly exhausted from the sfPESI emitter with complete exhaustion occurring by the point S5 (Figure 3c); at this point, while the ions for zolpidem cannot be observed, the spectrum is now exhibiting signals for the background phthalates with intense peaks observed at *m/z* 413, 391, and 149, which are still present in the spectrum when the analysis concludes at point S11 (Figure 3d). Figure S3 shows a single analysis where the extracted traces for the phthalate and the [ZPD + Na]^+^ ions are shown to illustrate the rapid separation mechanism.

**FIGURE 4:**
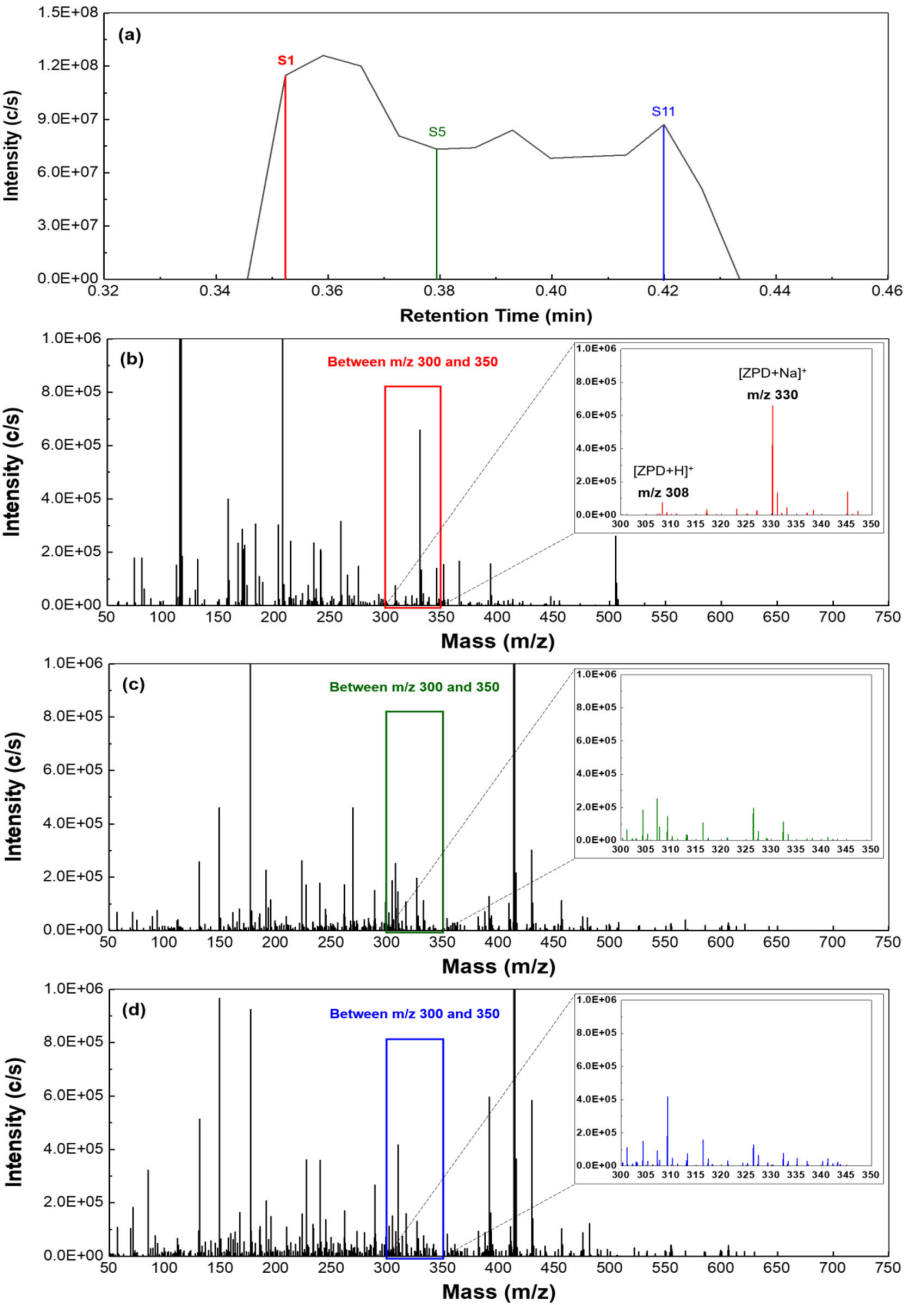
sfPESI‐MS analysis of a zolpidem‐doped fingerprint: (a) total ion chromatogram (TIC) obtained from a single analysis; (b) mass spectrum at scan S1 (inset showing region containing zolpidem ions); (c) mass spectrum at scan S5; and (d) mass spectrum at scan S11.

**FIGURE 5:**
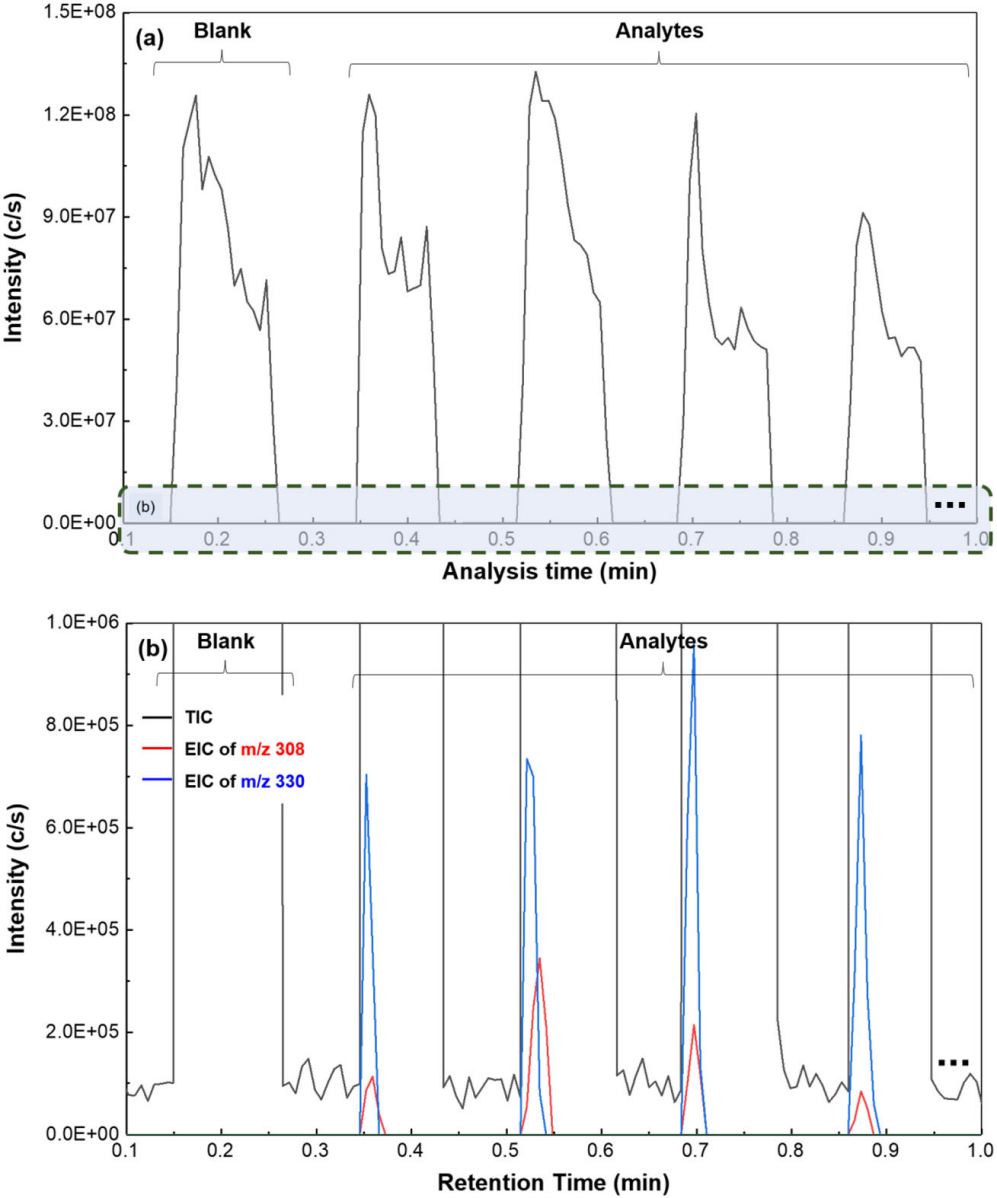
Total ion chromatograms (TIC) and extracted ion traces of zolpidem ions from the sfPESI‐MS analysis of blank and zolpidem‐doped fingerprint gel‐lifts: (a) TIC obtained from 1 blank and 4 zolpidem‐doped prints and (b) overlaid extracted ion chromatograms (EIC) for the [ZPD + H]+ ion (m/z 308.1755) and the [ZPD + Na]+ ion (m/z 330.1572) zoomed in to the scale shown in (a).

Extracted ion traces for the two detected zolpidem ions can be seen superimposed over the TIC trace in Figure 4. This figure shows a single measurement taken from a blank fingerprint gel lift and then four replicate measurements from a gel lift of fingerprint containing zolpidem residue. It can be clearly seen that no zolpidem is detected in the first blank print, while in the four other prints, the zolpidem ions are detected at the beginning of each replicate analysis with responses detected in the first four scans, while the high background levels persist until the end of the analysis. It can be seen in this figure that multiple replicate analyses can be acquired from test and blank prints within a short period of time with a blank and four replicates completed in approximately 1 min. While it is worth noting here that the sequential ionisation mechanism in this situation does not fully resolve the zolpidem ions from the complex matrix of the gel‐lifted fingerprint, the spectrum from point S1 shows significantly enhanced S:N ratio when compared with the averaged spectrum (Figure 5) and improves visualisation of the target analytes. The figure also shows that in this example with newly zolpidem‐doped fingerprints, each individual sfPESI analysis successfully detects the drug although there is a significant amount of variation between replicates. However, this is expected with a biological sample like the gel‐lifted fingerprint where distribution of zolpidem and other compounds across the print may not be completely even. Nevertheless, these data show that chemical information can be recovered reliably from gel‐lifted fingerprints using sfPESI and that the method warrants further investigation.

### 3.1 Investigation of three different surface materials

The [ZPD + H]^+^ ion at *m/z* 308.1755, [ZPD + Na]^+^ ion at *m/z* 330.1572, and [ZPD + K]^+^ ion at *m/z* 346.1336 (4.0‐ppm error) were analysed in full scan mode and monitored post‐acquisition to see if the surface influenced the recovery of zolpidem. The data presented in Table 2 show that the targeted molecular ions of ZPD were successfully detected from gel‐lifted fingerprints taken up from all three surface materials using sfPESI‐MS analysis, which demonstrates that the method effectively detect drug residues from both porous and nonporous surfaces. In all surfaces investigated, the [ZPD + Na]^+^ ion at *m/z* 330.1572 exhibited higher signal intensities and better detection precision (% RSD) than the [ZPD + H]^+^ and [ZPD + K]^+^ ions. The [ZPD + K]^+^ ion was only intermittently detected with it only being detected once in the 10 replicate measurements from the paper surface. The higher intensity observed for the [ZPD + Na]^+^ ion is expected with the presence of sodium ions in both the extraction solvent and the fingerprint itself. Of the three surfaces investigated, the lowest signal intensity was observed from the paper surface, which showed a response approximately three times lower than that obtained from glass slides, which may be due to the porous surface retaining some of the zolpidem. The brass surface shows an approximate 50% reduction in signal intensity; no copper adduct ions were observed in any of the spectra obtained from this surface. It is worth noting also that the %RSD is worse from paper (77% RSD) than both glass (30% RSD) and brass (36% RSD).

**TABLE 2:** Comparison of signal intensity and %RSD (*n* = 10) from gel‐lifted zolpidem‐doped prints recovered from glass, brass, and paper surfaces.

Surface materials	Intensity (c/s)^a^	Zolpidem (ZPD)		
		[ZPD + H]^+^	[ZPD + Na]^+^	[ZPD + K]^+^
		*m/z* 308.1755	*m/z* 330.1582	*m/z* 346.1336
Glass^b^	Average	3.7 × 10^4^	1.0 × 10^5^	7.2 × 10^3^
	STDEV^c^	15,847	29,834	2245
	% RSD^d^	42	30	31
Metal^e^	Average	3.1 × 10^4^	6.0 × 10^4^	7.7 × 10^3^
	STDEV	11,548	21,391	3045
	% RSD	38	36	40
Paper^f^	Average	1.8 × 10^4^	2.9 × 10^4^	8.2 × 10^3^
	STDEV	7775	22,596	‐
	% RSD	44	77	‐

^a^ Glass: glass microscope slides (W:25.4 × L:76.2 × T:1.2 mm, Sail Brand, China).

^b^ Paper: filter paper (Fisherbrand grade 601, Fisher Scientific, Loughborough, UK).

^c^ Metal: thin brass plate (W: 20.0 × L: 100.0 × T: 1.0 mm, Sweetnam and Bradley, Malmesbury, UK).

^d^ c/s: counts per second.

^e^ STDEV: standard deviation.

^f^ % RSD: per cent relative standard deviation.

### 3.2 Fingerprint depletion series analysis

**FIGURE 6:**
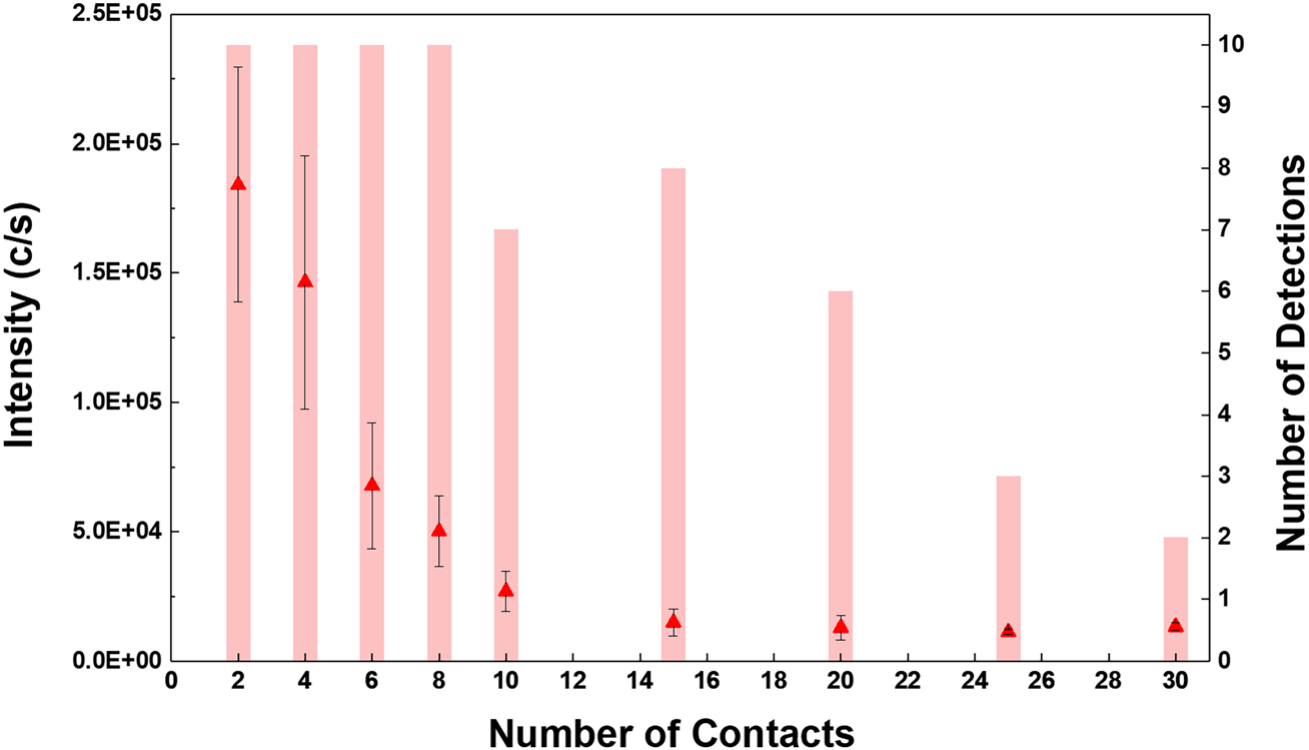
Graph showing average signal intensity (▲) and the number of detections (█) for the [ZPD + Na]^+^ ion (*m/z* 330.1572) from 10‐replicate depletion series of zolpidem‐doped fingerprint gel‐lifts analysed using sfPESI‐MS.

Glass slides were used as surface materials for all measurements, and standard gel‐lifter conditions as described earlier were used. Six replicate measurements were taken from each fingerprints on the gel‐lifter, and [ZPD + Na]^+^ ion at *m/z* 330.1572 was monitored as it provides the most intense and reproducible signal compared with the other zolpidem ions as shown in previous fingerprint analysis. sfPESI‐MS data were recorded on the second, fourth, sixth, eighth, 10th, 15th, 20th, 25th, and 30th prints in the depletion series and is presented in Figure 6 and Table S1. The [ZPD + Na]^+^ ion at *m/z* 330.1572 was detected in all of the depleted fingerprints analysed using sfPESI‐MS, although by the time 30 repeated contacts had been conducted only two out of 10 replicate measurements from the print gave a positive detection for ZPD as shown in Table S1. The signal intensity of [ZPD + Na]^+^ ion at *m/z* 330.1572 showed a considerable decrease as the number of contacts increased. This was clearly visible after only a small number of contacts and can be seen between the second and fourth contact of the gloved fingertip to the glass slide. This effect is not unexpected as more material from the gloved fingertips will be transferred in the first few contacts. However, despite this initial drop off in signal intensity, the sfPESI‐MS method reliably detected ZPD in each replicate measurement conducted after two, four, six, and eight touches had been made. After this point, the reliability of the technique in detecting ZPD in each measurement decreases with ZPD being detected in (on average) 7/10 measurements after 10, 15, and 20 touches and in only 3/10 measurements after 25 touches. When moving up to 30 consecutive contacts, only two analyses out of 10 positively detected ZPD. This shows that after 10 contacts, a larger number of replicates may be required to ensure ZPD is reliably detected. The speed of analysis with sfPESI here is a key advantage as conducting 10 replicates takes approximately 2 min and larger number of 20 or more could be possible especially if the system were automated to take samples from multiple points, which would improve the likelihood of detecting drug residues in gel‐lifted prints taken after large numbers of contacts.

## 4 CONCLUSIONS

The sfPESI‐MS method developed was capable of detecting zolpidem (ZPD) directly from gel‐lifted fingerprints in situ, a task that has not been possible with other methods developed in our laboratory such as using a thin layer chromatography (TLC) plate extractor or using direct thermal desorption. The gels are a difficult medium to work with; applying larger than microlitre volumes of solvent causes the gel to dissolve and foul the TLC plate extractor head, and elevated temperatures melt them, which causes a high level of background in thermally extracted samples. The sfPESI mechanism is shown to be beneficial for partially separating analytes from the complex gel and fingerprint background, and the small volume of liquid sampled and short sampling duration reduces damage to the gel. Three different surface materials (glass, metal, and paper) were investigated to determine the effect of deposition surface before taking up onto the gel‐lifter. The results of this experiment showed the surface material characteristics affected sample collection using gel‐lifter, although the targeted molecular ions ([ZPD + H]^+^ and [ZPD + Na]^+^ ions) were successfully detected from all fingerprints on the gel‐lifter taken up from the three different surface materials through sfPESI‐MS analysis. Lastly, a depletion series experiment from glass slides was conducted to determine whether drug residues could be detected of multiple physical interactions between surface and fingers. ZPD was detected on the gel‐lifter after 30 consecutive contacts; however, only two replicates out of 10 detected the drug residue at this point. The depletion series data show that drug residue could be detected with 100% detection efficiency after eight contacts with the detection efficiency dropping off after this point. The data presented show that sfPESI can successfully recover chemical information from gel‐lifters; this has significance in as the method could be used to investigate prints lifted from crime scenes and yield additional information that could shed light on the events around the crime. Gel‐lifted prints are frequently stored for many years after investigations, and the technique could be used to investigate cold cases where it could provide additional vital evidence. A significant advantage of sfPESI is that it can be readily coupled to different instruments. Combining it with a high‐resolution instrument like the orbitrap grants the ability to conduct scan mode analysis, which could be used to detect novel or unknown substances in the fingerprint. In combination with a tandem mass spectrometer like a triple quadrupole or Q‐Exactive Orbitrap, selected ion monitoring could be used to enhance sensitivity for targeted substances, which could further enhance the detection from depleted prints. Finally, because the sfPESI interface only requires a power supply and a supply of liquid solvent, the interface could readily be combined with a robust, low‐resolution instrument to enable remote use.

## Supporting information


**Figure S1.** Analysis workflow for depletion series analysis of latent fingerprints doped with zolpidem (ZPD) residue: (Step 1) wear gloves after washing hands, wear for 1 hour, remove, turn inside‐out and put back on; (Step 2) prepare a ZPD powder sample; (Step3) gently touch ZPD powder with a gloved finger, and generate 30 fingermarks containing ZPD residue; (Step 4) lift‐up the fingerprints using gel‐lifter (cutting by W: 20 x L: 60 mm); and (Step 5) direct analyse surfaces of the gel‐lifter depositing fingerprints containing ZPD residue (created with BioRender.com).
**Figure S2.** Averaged mass spectra obtained from the sfPESI‐MS of gel‐lifted fingerprints to show background: (a) full scan averaged mass spectrum from a single sfPESI‐MS analysis and (b) full scan averaged mass spectrum from a single sfPESI‐MS analysis zoomed in 10x on the y axis (the range from 0–2.4 × 10^7^ to 0–2.4 × 10^6^ c/s) (created with Origin19).
**Figure S3.** Total ion chromatogram (TIC) and extracted ion chromatograms (EIC) of a single analysis where the extracted traces for the phthalates and the [ZPD + Na]^+^ ions are shown to illustrate the rapid separation mechanism: (a) a TIC obtained a zolpidem doped print and (b) overlaid EIC for the phthalate ions (*m/z* 149 and *m/z* 391) and [ZPD + Na]^+^ ion (*m/z* 330) zoomed in approximately 100x on the y axis (the range from 0–1.5 × 10^8^ to 0–2.0 × 10^6^ c/s) (created with Origin19).
**Table S1.** Average signal intensity of [ZPD + Na]^+^ ion (at *m/z* 330.1572), %RSD precision and % Detection efficiency for depletion series experiments.
