# Supplementary material for: A direct analysis method using sheath flow probe electrospray ionisation‐mass spectrometry (sfPESI‐MS) to detect drug residues from fingerprint forensic gel lifts
Source: Drug Test Anal. 2024 Apr 8;17(1):152–62. doi: 10.1002/dta.3688 (PMC11729265; doi:10.1002/dta.3688)
Supplement: Supplementary file 1 — Figure S1. Analysis workflow for depletion series analysis of latent fingerprints doped with zolpidem (ZPD) residue: (Step 1) wear gloves after washing hands, wear for 1 hour, remove, turn inside‐out and put back on; (Step 2) prepare a ZPD powder sample; (Step3) gently touch ZPD powder with a gloved finger, and generate 30 fingermarks containing ZPD residue; (Step 4) lift‐up the fingerprints using gel‐lifter (cutting by W: 20 x L: 60 mm); and (Step 5) direct analyse surfaces of the gel‐lifter depositing fingerprints containing ZPD residue (created with BioRender.com). Figure S2. Averaged mass spectra obtained from the sfPESI‐MS of gel‐lifted fingerprints to show background: (a) full scan averaged mass spectrum from a single sfPESI‐MS analysis and (b) full scan averaged mass spectrum from a single sfPESI‐MS analysis zoomed in 10x on the y axis (the range from 0–2.4 × 10^7^ to 0–2.4 × 10^6^ c/s) (created with Origin19). Figure S3. Total ion chromatogram (TIC) and extracted ion chromatograms (EIC) of a single analysis where the extracted traces for the phthalates and the [ZPD + Na]+ ions are shown to illustrate the rapid separation mechanism: (a) a TIC obtained a zolpidem doped print and (b) overlaid EIC for the phthalate ions (m/z 149 and m/z 391) and [ZPD + Na]+ ion (m/z 330) zoomed in approximately 100x on the y axis (the range from 0–1.5 × 10^8^ to 0–2.0 × 10^6^ c/s) (created with Origin19). Table S1. Average signal intensity of [ZPD + Na]+ ion (at m/z 330.1572), %RSD precision and % Detection efficiency for depletion series experiments. [file DTA-17-152-s001.docx]

**A direct analysis method using Sheath Flow Probe Electrospray Ionization-Mass Spectrometry (sfPESI-MS) to detect drug residues from fingerprint forensic gel-lifts.**

Ayoung Kim,^a^ Paul F. Kelly, ^a^ Matthew A. Turner ^a^ and James C. Reynolds^a^*

^a^ Centre for Analytical Science, Department of Chemistry, Loughborough University, LE11 3TU, UK

* Corresponding author: [j.c.reynolds@lboro.ac.uk](mailto:j.c.reynolds@lboro.ac.uk)

**ELECTRONIC SUPPLEMENTARY INFORMATION**


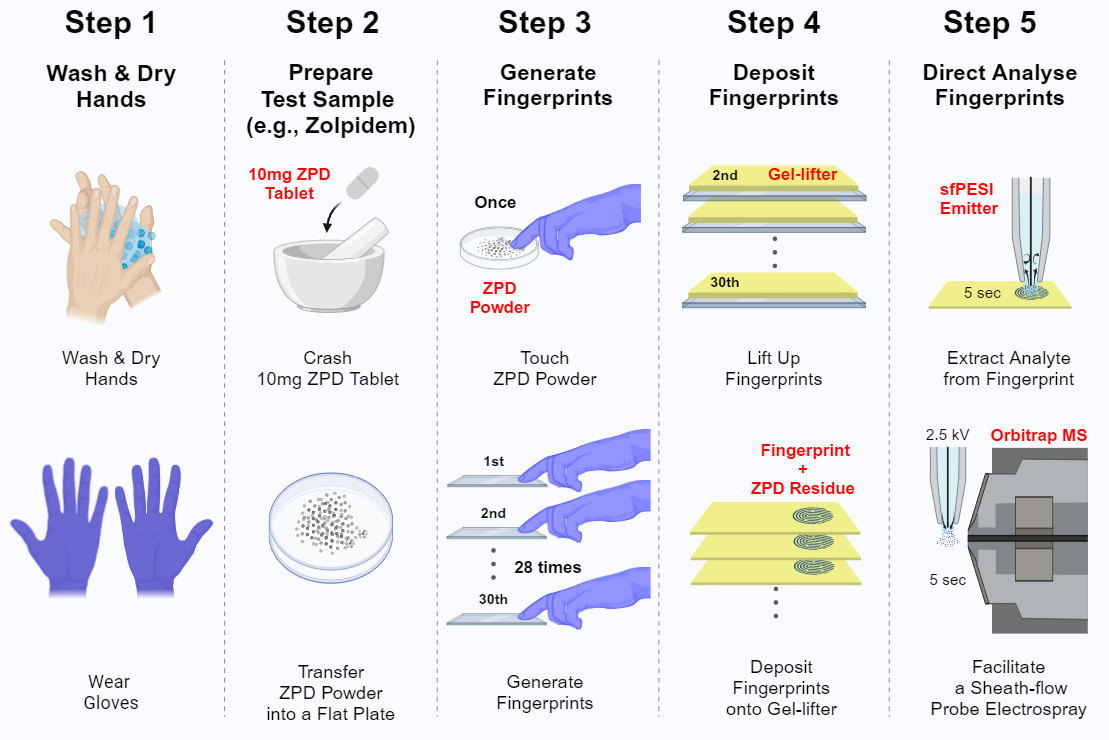


**Figure S1.** Analysis workflow for depletion series analysis of latent fingerprints doped with zolpidem (ZPD) residue: (Step 1) wear gloves after washing hands, wear for 1 hour, remove, turn inside-out and put back on; (Step 2) prepare a ZPD powder sample; (Step3) gently touch ZPD powder with a gloved finger, and generate 30 fingermarks containing ZPD residue; (Step 4) lift-up the fingerprints using gel-lifter (cutting by W: 20 x L: 60 mm); and (Step 5) direct analyse surfaces of the gel-lifter depositing fingerprints containing ZPD residue (created with BioRender.com).

**(a) Full scan averaged mass spectrum.**

**(b) Averaged mass spectrum zoomed into 10% on the y axis.**

**Figure S2.** Averaged mass spectra obtained from the sfPESI-MS of gel-lifted fingerprints to show background: (a) full scan averaged mass spectrum from a single sfPESI-MS analysis and (b) full scan averaged mass spectrum from a single sfPESI-MS analysis zoomed in 10x on the y axis (the range from 0-2.4$\times$10^7^ to 0-2.4$\times$10^6^ c/s) (created with Origin19).


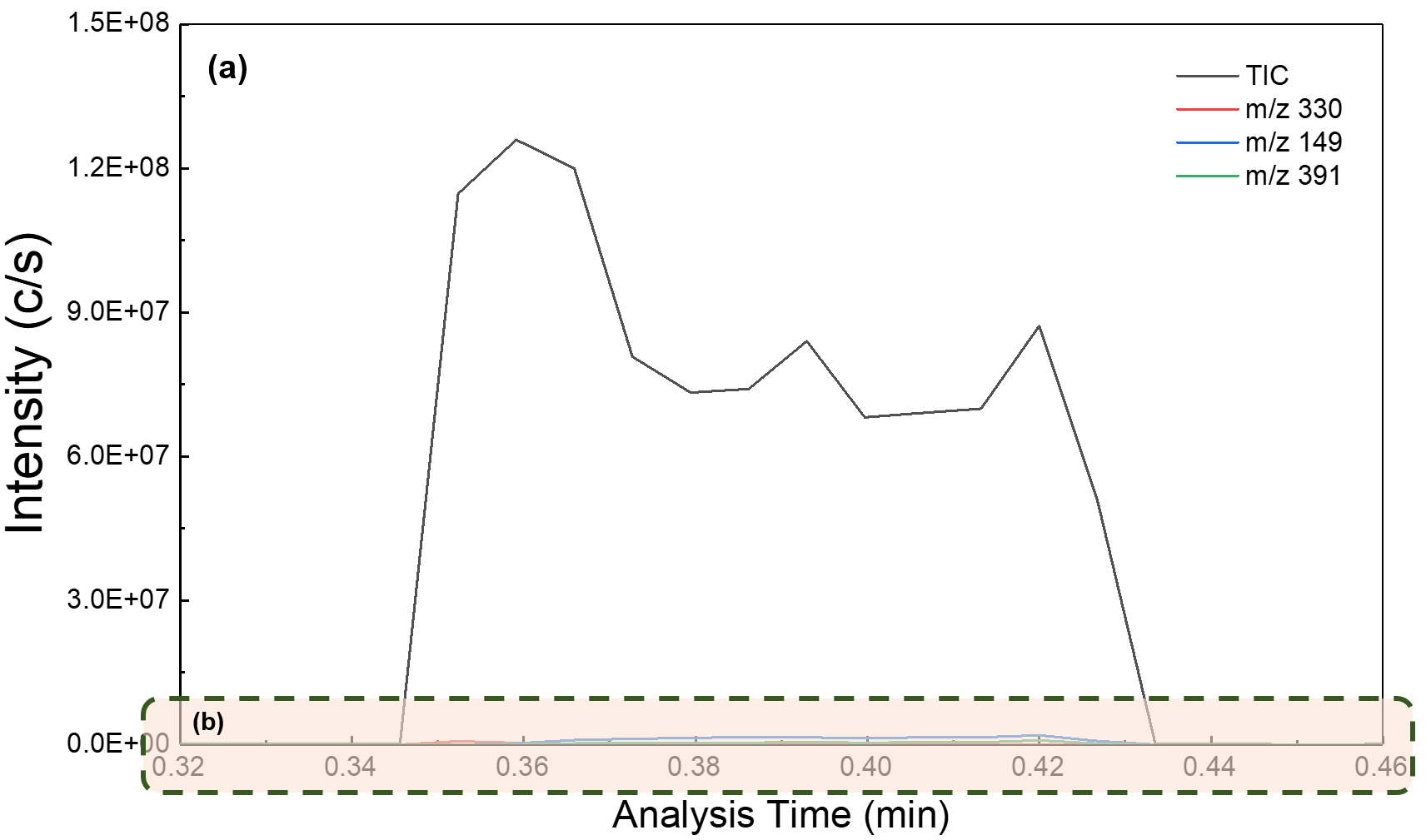


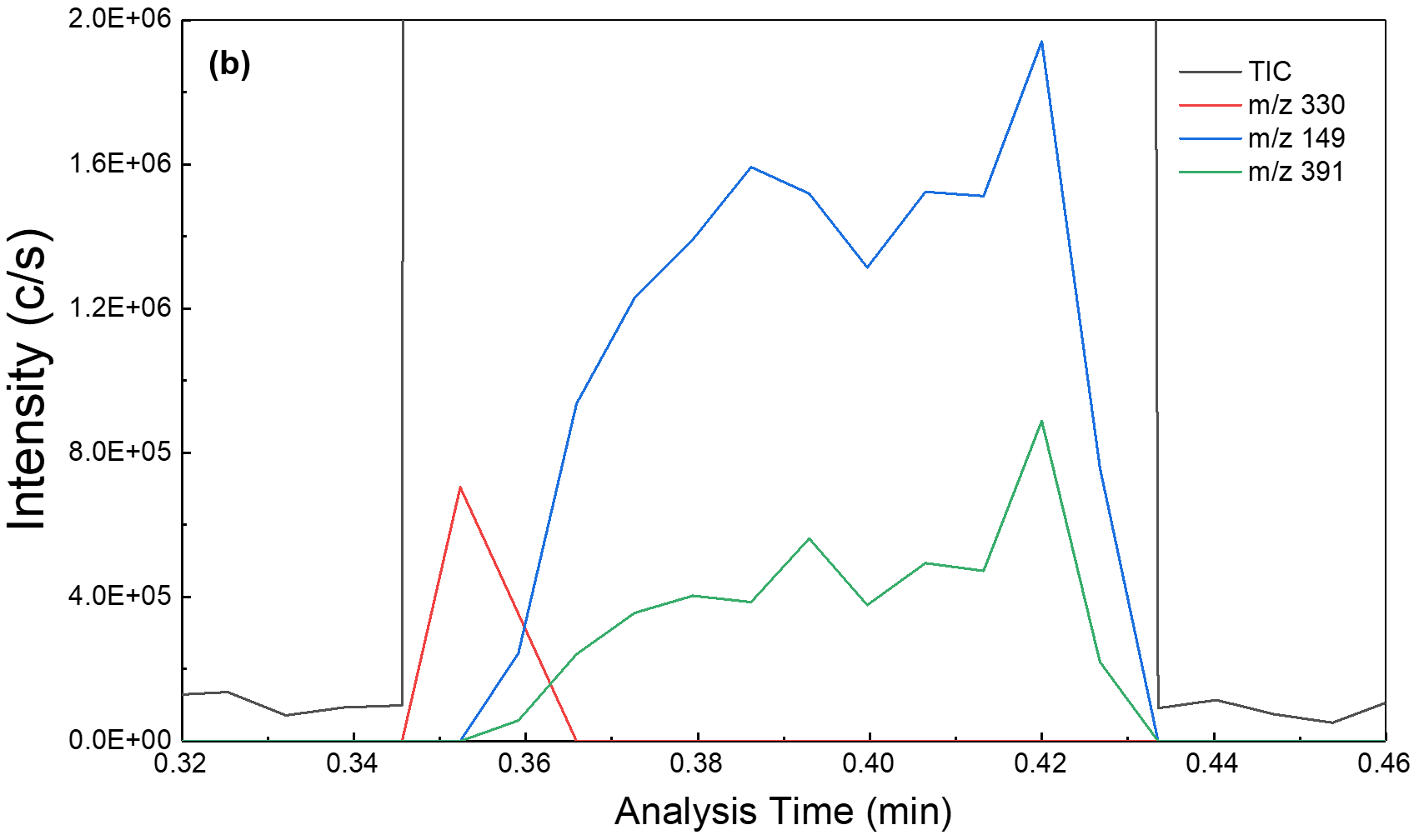


**Figure S3.** Total ion chromatogram (TIC) and extracted ion chromatograms (EIC) of a single analysis where the extracted traces for the phthalates and the [ZPD+Na]^+^ ions are shown to illustrate the rapid separation mechanism: (a) a TIC obtained a zolpidem doped print and (b) overlaid EIC for the phthalate ions (*m/z* 149 and *m/z* 391) and [ZPD+Na]^+^ ion (*m/z* 330) zoomed in approximately 100x on the y axis (the range from 0-1.5$\times$10^8^ to 0-2.0$\times$10^6^ c/s) (created with Origin19).

**Table S1.** Average signal intensity of [ZPD+Na]^+^ ion (at *m/z* 330.1572), %RSD precision and % Detection efficiency for depletion series experiments.

| Touch  Order | Intensity (c/s) ^a^ | | | Recovery ^d^  (The number of detection) |
| --- | --- | --- | --- | --- |
|  | Average | STDEV ^b^ | % RSD ^c^ |  |
| 2^nd^ | 1.8 x 10^5^ | 45,351 | 25 | 10 |
| 4^th^ | 1.5 x 10^5^ | 49,003 | 33 | 10 |
| 6^th^ | 6.8 x 10^4^ | 24,412 | 36 | 10 |
| 8^th^ | 5.0 x 10^4^ | 13,614 | 27 | 10 |
| 10^th^ | 2.7 x 10^4^ | 7,892 | 29 | 7 |
| 15^th^ | 1.5 x 10^4^ | 5,104 | 34 | 8 |
| 20^th^ | 1.3 x 10^4^ | 4,623 | 36 | 6 |
| 25^th^ | 1.1 x 10^4^ | 1,018 | 9 | 3 |
| 30^th^ | 1.3 x 10^4^ | 1,485 | 11 | 2 |

*a: c/s: counts per second,*

*b: STDEV: standard deviation,*

*c: % RSD: percent relative standard deviation, and*

*d: Recovery: the number of detection (“n“out of 10 replicates).*
